# Supplementary material for: Robust metabolic syndrome risk score based on triangular areal similarity
Source: PeerJ Comput Sci. 2024 Apr 25;10:e2015. doi: 10.7717/peerj-cs.2015 (PMC11057570; doi:10.7717/peerj-cs.2015)
Supplement: Supplemental Information 1 — Based on the current diagnostic criteria, case A had no risk factors, case B had three risk factors, and case B was diagnosed with MetS. However, based on the RMRS, case A was diagnosed with MetS at 0.560, but case B was at 0.458, which was not diagnosed as MetS. This opposite result occurred because in Case A, all factors were close to the threshold without the MetS factors exceeding the threshold, and in Case B, only three MetS factors slightly exceeded the threshold. N: the number of risk factors based on the current diagnostic criteria. Risk: risk score, F: Female. GL: Fasting glucose (ml/dl), WC: Waist circumference (cm), HDL: HDL-cholesterol (ml/dl), TG: Triglycerides (ml/dl), SBP: Systolic blood pressure (mm Hg), DBP: Diastolic blood pressure (mm Hg). [file peerj-cs-10-2015-s001.docx]

| **Case** | **N** | **Sex** | **Age** | **WC** | **SBP** | **DBP** | **GL** | **TG** | **HDL** | **Risk** |
| --- | --- | --- | --- | --- | --- | --- | --- | --- | --- | --- |
| A | 0 | F | 54 | 83 | 125 | 75 | 99 | 146 | 50 | 0.560 |
| B | 3 | F | 56 | 85 | 130 | 80 | 104 | 65 | 82 | 0.458 |
| **Thresholds** | | | | 85 | 130 | 85 | 100 | 150 | 50 | 0.547 |
