# Supplementary material for: Robust metabolic syndrome risk score based on triangular areal similarity
Source: PeerJ Comput Sci. 2024 Apr 25;10:e2015. doi: 10.7717/peerj-cs.2015 (PMC11057570; doi:10.7717/peerj-cs.2015)
Supplement: Supplemental Information 2 — N: NHANES, K: KoGES_HEXA, F: Female, M: Male, OH: Other Hispanic, NW: Non-Hispanic White, NB: Non-Hispanic Black, NA: Non-Hispanic Asian, OR: Other Race - Including Multi-Racial. [file peerj-cs-10-2015-s002.docx]

| Case | Dataset | Sex | Age | Race | Size(=n) | Prevalence |
| --- | --- | --- | --- | --- | --- | --- |
| 1 | N | All | All | All | 11,286 | 0.182 |
| 2 | K | All | All | All | 72,332 | 0.13 |
| 3 | N | F | All | All | 5,338 | 0.172 |
| 4 | N | M | All | All | 5,948 | 0.19 |
| 5 | K | F | All | All | 50,117 | 0.105 |
| 6 | K | M | All | All | 22,215 | 0.187 |
| 7 | N | F | 20< | All | 1,128 | 0.067 |
| 8 | N | F | 20-39 | All | 2,358 | 0.151 |
| 9 | N | F | 40-59 | All | 1,443 | 0.242 |
| 10 | N | F | 60> | All | 409 | 0.342 |
| 11 | N | M | 20< | All | 1,304 | 0.075 |
| 12 | N | M | 20-39 | All | 2,616 | 0.172 |
| 13 | N | M | 40-59 | All | 1,566 | 0.277 |
| 14 | N | M | 60> | All | 462 | 0.32 |
| 15 | K | F | 40-59 | All | 43,120 | 0.088 |
| 16 | K | F | 60> | All | 6,997 | 0.207 |
| 17 | K | M | 40-59 | All | 17,407 | 0.187 |
| 18 | K | M | 60> | All | 4,808 | 0.184 |
| 19 | N | F | All | OH | 1,044 | 0.236 |
| 20 | N | F | All | NW | 509 | 0.187 |
| 21 | N | F | All | NB | 1,957 | 0.166 |
| 22 | N | F | All | NA | 1,163 | 0.152 |
| 23 | N | F | All | OR | 665 | 0.116 |
| 24 | N | M | All | OH | 1,260 | 0.237 |
| 25 | N | M | All | NW | 541 | 0.2 |
| 26 | N | M | All | NB | 2,167 | 0.216 |
| 27 | N | M | All | NA | 1,317 | 0.121 |
| 28 | N | M | All | OR | 663 | 0.145 |
