# Supplementary figures and images for: Robust metabolic syndrome risk score based on triangular areal similarity
Source: PeerJ Comput Sci. 2024 Apr 25;10:e2015. doi: 10.7717/peerj-cs.2015 (PMC11057570; doi:10.7717/peerj-cs.2015)

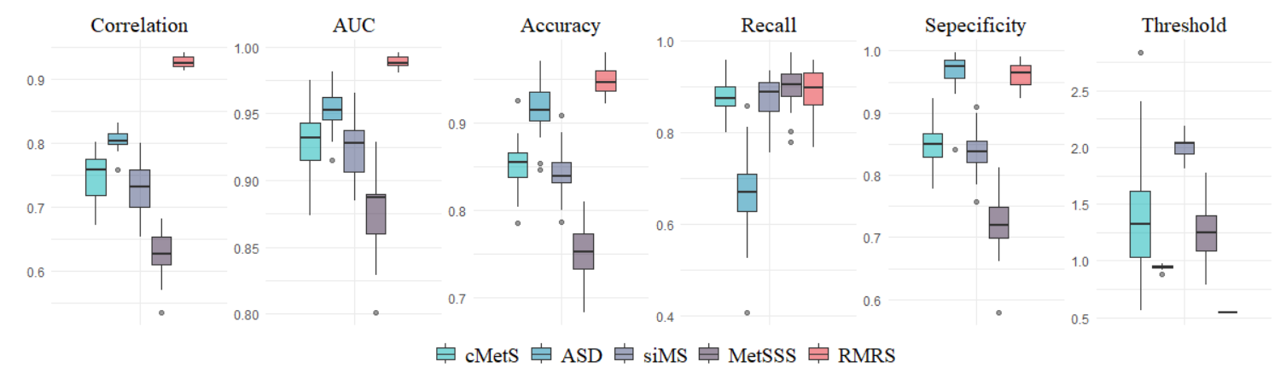

Supplement: Supplemental Information 3 — The performance was measured for each 28 case and the distribution was compared. [file peerj-cs-10-2015-s003.png]
